# Prevalence of postterm births and associated maternal risk factors in China: data from over 6 million births at health facilities between 2012 and 2016

**DOI:** 10.1038/s41598-018-36290-7

**Published:** 2019-01-22

**Authors:** Kui Deng, Yan Huang, Yanping Wang, Jun Zhu, Yi Mu, Xiaohong Li, Aiyun Xing, Zheng Liu, Mingrong Li, Xiaodong Wang, Juan Liang

**Affiliations:** 1 National Office for Maternal and Child Health Surveillance of China, West China Second University Hospital, Sichuan University, Chengdu, Sichuan China; 2 Nursing Department, West China Second University Hospital, Sichuan University, Chengdu, Sichuan China; 3 Department of Obstetrics, West China Second University Hospital, Sichuan University, Chengdu, Sichuan China; 4 Key Laboratory of Birth Defects and Related Diseases of Women and Children (Sichuan University), Ministry of Education, Chengdu, Sichuan China

## Abstract

Postterm births are associated with an increased risk of adverse perinatal outcomes, but few studies have investigated the epidemiological characteristics of postterm births. We aimed to estimate the prevalence of postterm births and examine the potential association between maternal sociodemographic and obstetric characteristics and postterm births. Data were collected from China’s National Maternal Near Miss Surveillance System, 2012–2016. A logistic regression was used to assess the association between sociodemographic and obstetric characteristics and postterm births. A Poisson regression was used to determine the crude and adjusted trends of postterm births over time across regions. Among the 6,240,830 singleton births with gestational periods of 37 weeks or longer, 1.16% were postterm. The prevalence of postterm births was significantly higher in the western region and among mothers who delivered at a level ≤2 hospital, had a lower education, or were younger. A reduced risk of postterm births was observed among primiparous women, mothers who previously had a caesarean section, mothers with pregnancy complications, and mothers with ten or more antenatal visits. The risk of postterm births decreased as the number of antenatal visits increased. The overall postterm birth rates significantly decreased from 1.49% in 2012 to 0.70% in 2016. The postterm birth rates were markedly reduced in the east, central, and west regions, and the rate of the decrease was greater in the east than in the west. Furthermore, substantial decreases were observed across regions in 2014 and 2016. In conclusion, multiple sociodemographic and obstetric factors are associated with the prevalence of postterm births. A significant decreasing trend in postterm birth rates was observed in China.

## Introduction

Gestational age is among the most important determinants of perinatal outcomes, but studies have focused more on preterm births (<37 completed gestational weeks) and less on the understanding and prevention of postterm births^1–3^. According to the World Health Organization, postterm birth is defined as birth that occurs after 42 completed weeks (294 days) of gestation^4^. Postterm births are associated with an increased risk of maternal complications, including postpartum hemorrhage, dystocia, and caesarean deliveries^5–7^. Furthermore, babies who are born postterm have increased perinatal and neonatal mortality and morbidity (e.g., meconium aspiration, fetal distress, and traumatic injury)^7–10^.

As previously reported, the prevalence of postterm births ranges from 1–10% worldwide, but large differences exist between and within countries depending on the diversity of the populations studied and variations in obstetric practices^11–13^. The etiology of postterm births is largely unknown, but multiple risk factors are associated with the prevalence of postterm births, including genetic factors^14,15^, maternal age^16–19^, education^16,18,19^, pregnancy body mass index^17–20^, primiparity^17–19,21^, previous postterm pregnancy^16,22^, and maternal obstetric complications^19^. Among these factors, some (e.g., maternal age, education, and primiparity) have shown conflicting results. However, epidemiological studies investigating postterm births are relatively limited. In particular, the availability of nationwide data regarding postterm births in China is limited.

The present study aims to estimate the prevalence of postterm births using the largest and most updated dataset from the National Maternal Near Miss Registry in China. We also examined the potential association between maternal sociodemographic and obstetric characteristics and postterm births.

## Materials and Methods

### Data sources and study population

The data in this study were derived from China’s National Maternal Near Miss Surveillance System (NMNMSS) between January 1, 2012, and December 31, 2016. The NMNMSS was established in October 2010 and covers 438 health facilities at the county level and above across 326 urban districts and rural counties based on the National Maternal and Child Mortality Surveillance System. The sampling methods have been described in detail elsewhere^21,22^. Within each sampled district/county, two health facilities (or one facility if only one was available) with more than 1,000 deliveries per year were randomly selected. Because health facilities with the necessary number of births were mostly located in urban areas, large hospitals in urban districts were oversampled, particularly in the central and western regions. This study was approved by the Ethic Review Committee of Sichuan University. The requirement for obtaining informed consent was waived because of the retrospective design of this study. This study was carried out in accordance with the principles of the Declaration of Helsinki.

The maternal sociodemographic and obstetric characteristics of all pregnant and postpartum women who were admitted to the obstetric departments at each of the surveillance facilities were prospectively collected from the time of admission until discharge. The doctors responsible for patient care were trained to complete a specially designed data collection form for each patient. The data from each member hospital were entered into a web-based data management system that was centralized at the National Office for Maternal and Child Health Surveillance of China. County-, municipal-, and provincial-level Maternal and Child Health Hospital staff members visited all selected facilities once or twice a year and performed data quality management. The National Office for Maternal and Child Health Surveillance also visited six to eight randomly selected hospitals in each province once a year to evaluate the quality of the records. At each level, a panel of senior health professionals evaluated the completeness, accuracy, and timeliness of the data. If errors exceeded a predefined standard (e.g., if obstetric complications were under-reported by more than 5%, maternal deaths were under-reported by more than 1%, and maternal near misses were under-reported by more than 5%), the surveillance facilities were required to re-examine all data.

### Data definitions

In our analysis, postterm birth was defined as labor at or after 42 weeks of gestation. The gestational age is generally estimated on the basis of the last menstrual period or prenatal ultrasound if the date of the last menstrual period is unknown. The following sociodemographic characteristics were considered in our analysis: region (eastern, central, and western), hospital level (unknown, level 1, level 2, and level 3), maternal age (<20, 20–24, 25–29, 30–34, 35–39, 40–44, and 45–49 years), maternal education (illiteracy, primary school, middle school, high school, and college or higher), and marital status (married or single/widowed/divorced). The hospital level (levels 1–3) was defined based on the number of beds (level 3 hospitals have more beds than level 1 and level 2 hospitals), categories of clinical departments, number of medical staff, type and quantity of equipment, and hospital funding. The obstetric variables related to postterm birth included the number of antenatal visits, parity, history of a caesarean section, and maternal complications. Maternal complications were classified hierarchically into mutually exclusive categories of direct obstetric complications, medical diseases, and none of the above. Direct obstetric complications included uterine rupture, placenta Previa, abruptio placentae, unspecified antepartum hemorrhage, pre-eclampsia, eclampsia, HELLP syndrome, or any fetal malpresentation (breech, shoulder, or other). Medical diseases included heart disease, embolism/thrombophlebitis, hepatic disease, severe anemia (hemoglobin <70 g/L), renal disease (including urinary tract infections), lung disease (including upper respiratory tract infections), HIV/AIDS, connective tissue disorders, gestational diabetes mellitus, and cancer.

### Statistical analysis

We explored the distribution of postterm births according to different maternal sociodemographic and obstetric characteristics. A logistic regression was used to examine the strength of the association between the sociodemographic and obstetric characteristics and postterm births considering the sampling strategy and the clustering of births within hospitals. The data were weighed against the probability that each individual could be included in the sample from each region or rural or urban area to account for sampling issues, as described previously^21^. Crude odds ratios (ORs) with 95% confidence intervals (CIs) and ORs adjusted for sociodemographic and obstetric characteristics are reported.

Additionally, we report the time trends of the postterm birth rates using a Poisson regression with a robust variance estimator to estimate the crude and adjusted relative risks and 95% CIs of postterm births by region and year. To examine whether the postterm birth trends over time were similar across regions, the *p-*values of the interaction terms (region and year) were calculated by comparing the models with and without interaction terms using a likelihood ratio test. The statistical analyses were performed using STATA 14.2 (Stata Corporation, College Station Texas, USA).

## Results

**Figure 1:**
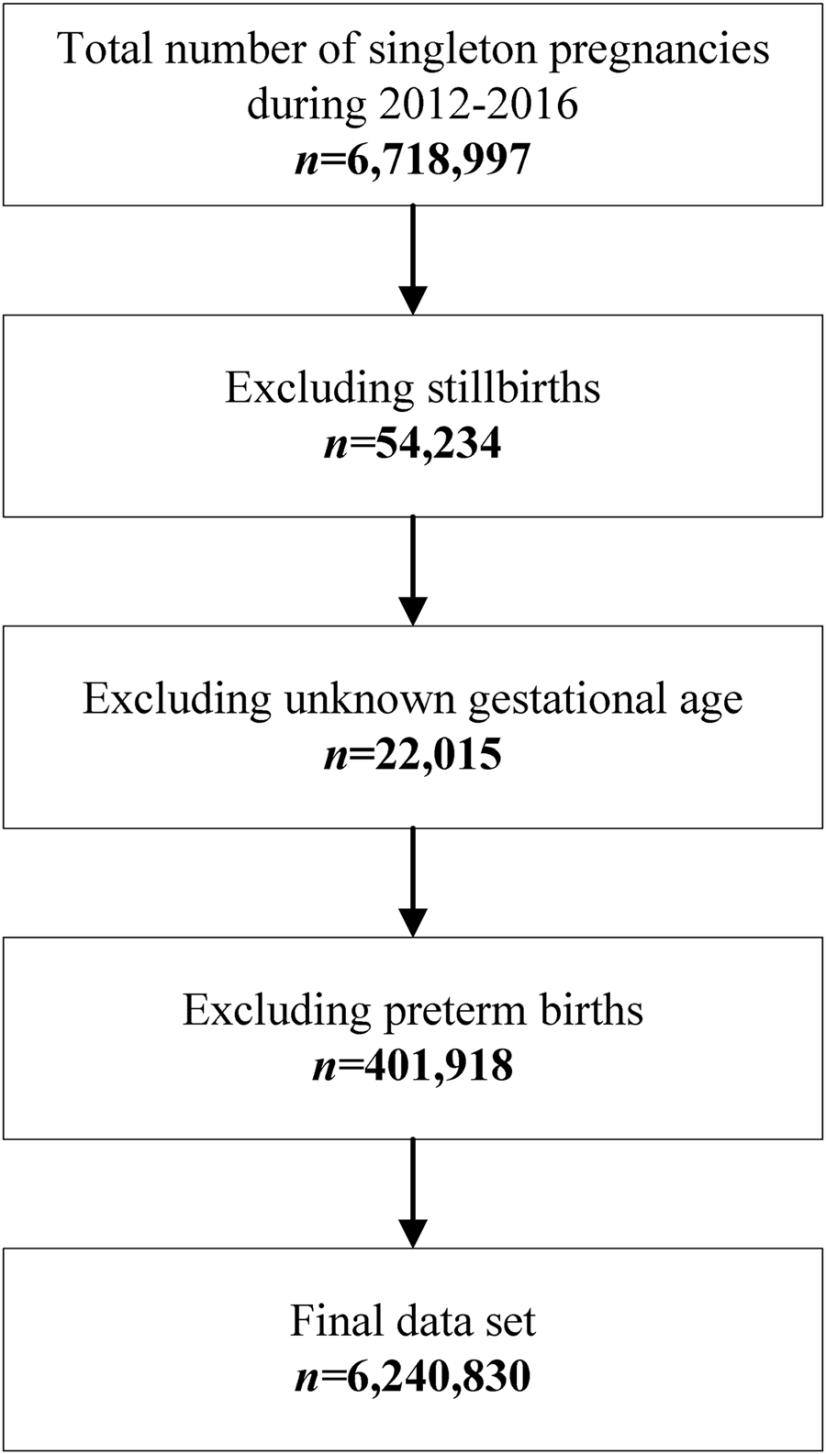
Study sample design from the National Maternal Near Miss Surveillance System in China between 2012 and 2016.

There were 6,718,997 singleton births between January 1, 2012, and December 31, 2016 according to the NMNMSS. We excluded 54,234 stillbirths, 22,015 births with unknown gestational age, and 401,918 preterm births (birth before 37 completed weeks of gestation). The final dataset included 6,240,830 singleton births at ≥37 gestational weeks (Fig. 1). Of these births, 61,559 births were delivered at or after 42 completed weeks of gestation, resulting in a weighted postterm birth rate of 1.16% (95% CI 1.15–1.17). Table 1 shows the maternal sociodemographic characteristics of all births. Most births were delivered at level 2 or level 3 hospitals, most women had attended school and completed their primary education, and nearly all women were married. Most women gave birth between the ages of 20 and 40 years.

**Table 1:** Associations between maternal sociodemographic characteristics and postterm births in China, 2012–2016.

Characteristics	Number of births*	Postterm per 100 births^†^	Crude OR (95% CI)^†‡^	Adjusted OR (95% CI)^†‡☦^
Total	6,240,830	1.16 (61,559)		
**Region**				
East	1,818,514 (29.14)	0.74 (11,530)	1.00	1.00
Central	2,485,477 (39.83)	1.14 (24,393)	1.56 (1.13–2.14)	1.15 (0.93–1.43)
West	1,936,839 (31.03)	1.68 (25,636)	2.31 (1.67–3.20)	1.56 (1.26–1.93)
**Hospital level**				
Unknown	318,110 (5.10)	1.96 (5,018)	4.25 (2.71–6.67)	2.04 (1.51–2.74)
Level 1	438,404 (7.02)	1.52 (6,508)	3.28 (2.41–4.47)	1.46 (1.11–1.92)
Level 2	3,026,314 (48.49)	1.40 (38,755)	3.03 (2.38–3.85)	1.64 (1.37–1.97)
Level 3	2,458,002 (39.39)	0.47 (11,278)	1.00	1.00
**Maternal age (years)**				
<20	181,026 (2.90)	2.59 (4,462)	3.35 (2.99–3.76)	1.86 (1.73–2.00)
20–24	1,391,844 (22.30)	1.66 (20,954)	2.13 (1.96–2.31)	1.51 (1.44–1.58)
25–29	2,631,150 (42.16)	0.96 (21,279)	1.23 (1.17–1.28)	1.16 (1.12–1.21)
30–34	1,298,458 (20.81)	0.79 (8,451)	1.00	1.00
35–39	439,260 (7.04)	0.80 (2,947)	1.02 (0.97–1.07)	0.90 (0.86–0.94)
40–44	88,787 (1.42)	0.91 (685)	1.17 (1.04–1.30)	0.88 (0.79–0.97)
45–49	5,421 (0.09)	1.08 (52)	1.37 (1.02–1.85)	0.86 (0.64–1.15)
**Maternal education**				
Illiteracy	30,977 (0.50)	2.99 (844)	8.32 (6.77–10.21)	4.24 (3.63–4.95)
Primary school	203,444 (3.26)	2.52 (4,939)	6.97 (5.92–8.22)	3.50 (3.09–3.98)
Middle school	2,161,724 (34.64)	1.70 (34,900)	4.67 (4.04–5.40)	2.54 (2.26–2.85)
High school	1,660,009 (26.60)	0.95 (13,702)	2.58 (2.26–2.95)	1.72 (1.55–1.92)
College or higher	2,052,411 (32.89)	0.37 (6,796)	1.00	1.00
**Marital status**				
Married	6,151,421 (98.57)	1.15 (60,148)	1.00	1.00
Single, widowed, or divorced	88,171 (1.41)	1.72 (1,406)	1.50 (1.29–1.75)	0.90 (0.78–1.05)

Data shown in parentheses are % of births, numbers of postterm births, and 95% CIs. CIs: confidence intervals.

*May not equal the total number of cases due to missing values of some characteristics.

^†^Adjusted for the sampling distribution of the population.

^‡^Adjusted for the clustering of births within hospitals.

^☦^Adjusted for all other variables in the table and maternal obstetric characteristics (antenatal visits, parity, history of caesarean sections, and maternal complications).

The associations between the maternal sociodemographic characteristics and postterm births are presented in Table 1. Compared with the women in the east, the women located in the west and central regions were more likely to have postterm births. The highest postterm birth rate occurred in the west. The association between maternal education and postterm births was particularly strong. Women who were illiterate were more than four times more likely to have a postterm birth than women who had completed college or higher, whereas women who had only attended primary school had a three times greater risk. A U-shape distribution was observed between the maternal ages and postterm births. Mothers aged <20 years, 20–24 years, 40–44 years, and 45–49 years had a higher risk of postterm births than mothers aged 30–34 years in the crude analysis. However, a slight risk reduction was found between mothers aged 35–39 years and 40–44 years and postterm births and no statistically significant relationship was found between mothers aged 45–49 years and postterm births after adjusting for the other sociodemographic factors and maternal obstetric characteristics. Additionally, compared with level 3 hospitals, the postterm birth rate at hospitals with unknown levels and level 1 and level 2 hospitals was higher. Marital status seemed to be associated with postterm births in the crude analysis, but this effect was not statistically significant after adjusting for the other sociodemographic factors and maternal obstetric characteristics.

Table 2 shows the association between the maternal obstetric characteristics and postterm births. The postterm birth rate decreased as the number of antenatal visits increased. Women with no antenatal visits were approximately twice more likely to have a postterm birth than women with ten or more visits. Women with direct obstetric complications or medical diseases had a lower than ~30% decrease in the risk of postterm births compared to those with no complications. Women with a history of a caesarean section had an approximately 50% lower risk of postterm birth than those with no previous caesarean sections. Additionally, a slight reduction in the risk of postterm births was observed in primiparous women compared with parous women.

**Table 2:** Associations between maternal obstetric characteristics and postterm births in China, 2012–2016.

Characteristics	Number of births*	Postterm per 100 births^†^	Crude OR (95% CI)^†‡^	Adjusted OR (95% CI)^†‡☦^
**Total**	6,240,830	1.16 (61,559)		
**Antenatal visits**				
None	88,515 (1.42)	1.92 (1,615)	3.81 (3.08–4.72)	1.97 (1.67–2.33)
1–3	459,785 (7.37)	1.90 (8,084)	3.78 (3.06–4.65)	1.77 (1.51–2.09)
4–6	1,982,925 (31.77)	1.58 (28,368)	3.13 (2.61–3.75)	1.60 (1.39–1.85)
7–9	1,819,596 (29.16)	0.95 (14,633)	1.87 (1.60–2.18)	1.26 (1.12–1.41)
≥10	1,736,589 (27.83)	0.51 (7,649)	1.00	1.00
**Parity**				
0	3,762,340 (60.29)	1.10 (34,269)	0.88 (0.81–0.95)	0.90 (0.85–0.94)
≥1	2,476,413 (39.68)	1.25 (27,250)	1.00	1.00
**History of caesarean sections**				
Yes	830,988 (13.32)	0.60 (4,254)	0.47 (0.44–0.51)	0.47 (0.44–0.52)
No	5,403,791 (86.59)	1.25 (57,224)	1.00	1.00
**Maternal complications**				
Direct obstetric complications	339,377 (5.44)	0.69 (1,925)	0.56 (0.51–0.62)	0.64 (0.59–0.70)
Medical diseases	386,417 (6.19)	0.56 (1,785)	0.45 (0.39–0.52)	0.72 (0.65–0.81)
None of the above	5,515,036 (88.37)	1.23 (57,849)	1.00	1.00

Data shown in parentheses are % of births, numbers of postterm birth, and 95% CIs. CIs: confidence intervals.

*May not equal the total number of cases due to missing values of some characteristics.

^†^Adjusted for the sampling distribution of the population. ^‡^Adjusted for the clustering of births within hospitals.

^☦^Adjusted for maternal location, hospital level, maternal ages, and maternal education.

Table 3 shows the trends of postterm birth rates among the regions over time. The overall postterm birth rate declined from 1.49% in 2012 to 0.70% in 2016 (crude relative risk = 0.47, 95% CI: 0.43–0.51). Adjusting the time trend for the maternal sociodemographic and obstetric characteristics increased the relative risk to 0.59 (95% CI: 0.55–0.65), suggesting that the postterm birth rate decreased by 41% between 2012 and 2016. The crude postterm birth rates also decreased within the regions during the study periods. The rate of decline was greater in the east (crude relative risk = 0.40, 95% CI: 0.34–0.47) than in the west (crude relative risk = 0.52, 95% CI: 0.44–0.61) (interaction between region and year, P < 0.001). Regardless of the region, substantial declines in the postterm birth rates were observed in 2014 and 2016. Adjusting for the maternal sociodemographic and obstetric characteristics increased the relative risks, but similar time trends were observed across the regions.

**Table 3:** Time trends of postterm birth rates among all births by region in China, 2012–2016.

Region	2012	2013	2014	2015	2016	*P*-value (interaction)
**Weighted postterm birth rate (%) (No of postterm births, % of postterm births)** ^**†**^						
Total	1.49 (15,329, 100.0)	1.44 (14,257, 100.0)	1.16 (13,091, 100.0)	1.08 (10,767, 100.0)	0.70 (8,115, 100.0)	
East	0.99 (3,042, 19.84)	0.95 (2,692, 18.88)	0.75 (2,589, 19.78)	0.64 (1,861, 17.28)	0.40 (1,346, 16.59)	—
Central	1.49 (6,161, 40.19)	1.40 (5,665, 39.73)	1.11 (5,039, 38.49)	1.08 (4,300, 39.94)	0.68 (3,228, 39.78)	
West	2.08 (6,126, 39.96)	2.03 (5,900, 41.38)	1.73 (5,463, 41.73)	1.58 (4,606, 42.78)	1.08 (3,541, 43.64)	
**Crude relative risk (95% CI)** ^**†‡**^						
Total	1.00	0.96 (0.93–1.00)	0.78 (0.74–0.81)	0.72 (0.67–0.78)	0.47 (0.43–0.51)	
East	1.00	0.95 (0.89–1.03)	0.76 (0.70–0.82)	0.65 (0.57–0.73)	0.40 (0.34–0.47)	0.000
Central	1.00	0.94 (0.89–0.99)	0.74 (0.70–0.79)	0.72 (0.66–0.79)	0.46 (0.41–0.50)	
West	1.00	0.98 (0.92–1.04)	0.83 (0.76–0.91)	0.76 (0.66–0.87)	0.52 (0.44–0.61)	
**Adjusted relative risk (95% CI)** ^**†‡☦**^						
Total	1.00	0.99 (0.96–1.02)	0.85 (0.82–0.89)	0.81 (0.76–0.86)	0.59 (0.55–0.65)	
East	1.00	1.00 (0.94–1.07)	0.86 (0.80–0.93)	0.75 (0.67–0.84)	0.54 (0.46–0.63)	0.000
Central	1.00	0.98 (0.93–1.03)	0.83 (0.79–0.88)	0.84 (0.77–0.92)	0.59 (0.53–0.66)	
West	1.00	0.99 (0.93–1.05)	0.86 (0.79–0.94)	0.80 (0.71–0.91)	0.62 (0.52–0.73)	

CIs: confidence intervals.

^†^Adjusted for the sampling distribution of the population. ^‡^Adjusted for the clustering of births within hospitals.

^☦^Adjusted for the hospital level, maternal ages, maternal education, antenatal visits, parity, history of caesarean sections, and maternal complications.

**Figure 2:**
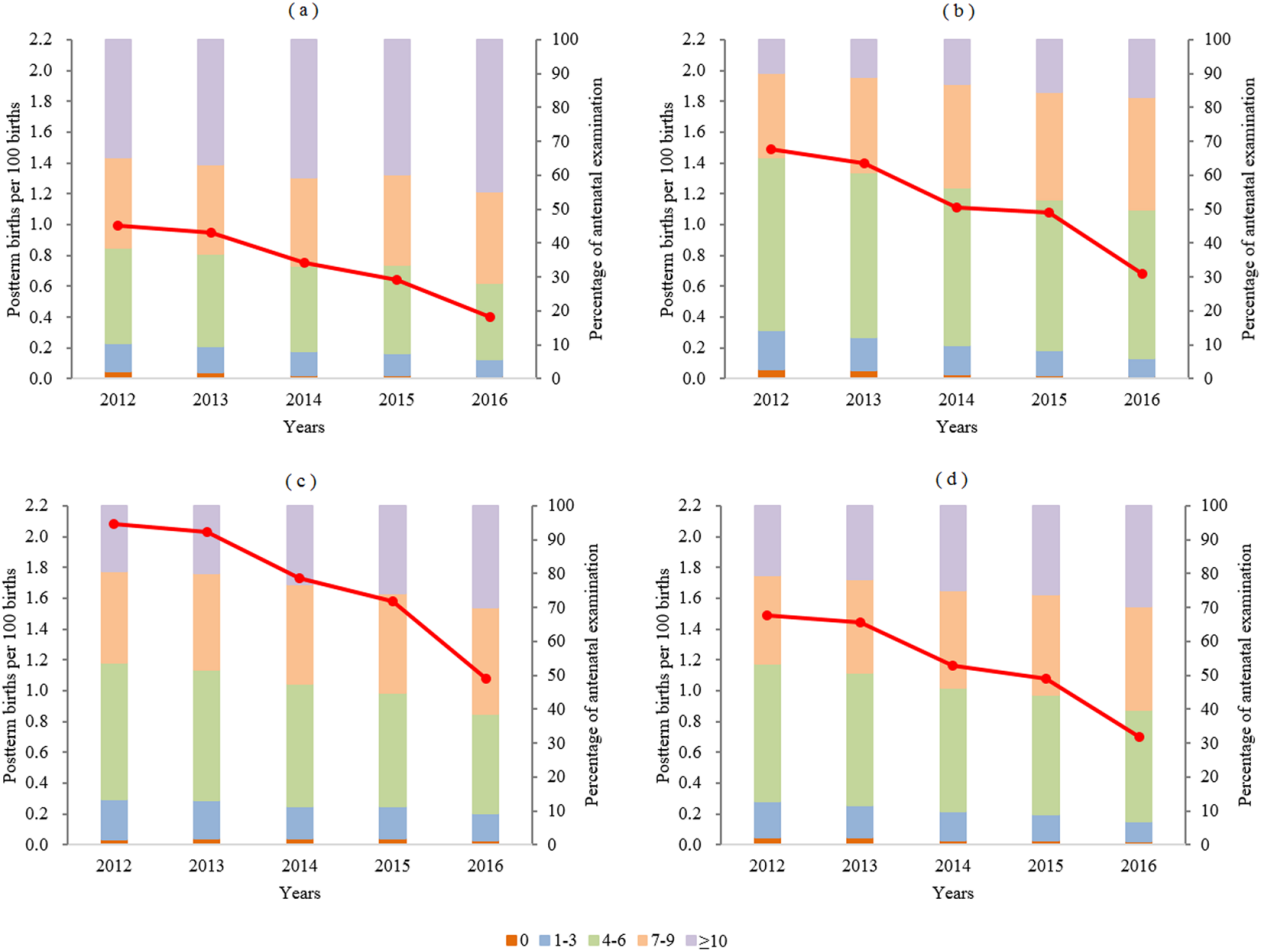
Changes in postterm birth rates and percentage of antenatal visits by region in China, 2012–2016. East (**a**), central (**b**), west (**c**), and national (**d**). The rates of postterm births were adjusted for the sampling distributions of the population. The red solid line represents the time trend of postterm birth rates. For each region, the decline trend of postterm birth rates is statistically significant (P < 0.0001).

Figure 2 presents the changes in postterm birth rates and percentages of antenatal visits from 2012 to 2016. During the five-year study period, the overall proportions of women with no antenatal visits and those with 1–3 antenatal visits significantly decreased from 2.2% and 10.7% in 2012 to 0.9% and 6.0% in 2016, respectively (P < 0.0001 for both), whereas the proportions of women with 7–9 antenatal visits and 10 or more antenatal visits increased from 26.2% and 20.5% in 2012 to 30.7% and 29.6% in 2016, respectively (P < 0.0001 for both). Similar trends were observed across the regions over time. Regardless of the region, the yearly postterm birth rates significantly decreased as the proportions of women with no more than three antenatal visits decreased and the proportions of women with more than six antenatal visits increased.

## Discussion

The present study shows that based on the largest dataset from the Chinese Maternal Near Miss Registry, the overall prevalence of postterm births among singleton live births at ≥37 weeks of gestation is 1.16%. We list the sociodemographic factors and obstetric characteristics that are associated with postterm births. Furthermore, we find that the prevalence of postterm births is significantly decreasing in China.

Previous studies have reported large variations in postterm birth rates among countries. In Europe, a study investigating thirteen European countries found that the postterm birth rates range from 0.4% (Austria and Belgium) to over 7% (Denmark and Sweden)^12^. In the United States and Canada, the postterm birth rates range from 1.0% to 2.5%^23^. The present study estimates that the weighted postterm birth rate in China between 2012 and 2016 was 1.16%, which may be overestimated due to the exclusion of preterm births and stillbirths. This estimated rate is similar to that in France^11^, Canada^23^, and the United States^23^ but lower than that in most European countries^12^. The inconsistency of these results may be a consequence of the sampled population and obstetric practices, including different policies for labor induction and methods of assessing the gestational age. Measuring the gestational age using an ultrasound scan in early pregnancy than based on the last menstrual period and routine labor induction before 42 weeks can reduce the frequency of postterm births^13,24^. Many national scientific societies have published individual guidelines for prolonged pregnancy management based on local health systems^11,13,25^. The Chinese Society of Obstetrics and Gynecology updated the clinical guidelines for cervical ripening and labor induction in 2014^26^. However, the current policies for prolonged pregnancy management are concordant among countries.

Generally, significant variations in economic, cultural, and educational developments exist between and within regions (eastern, central, and western) because of the non-uniform rates of development in China. The eastern area is more developed than the central and western areas. Therefore, the level of antenatal care and awareness of pregnancy healthcare among women in the east may be higher than those among women in the west. Level 3 hospitals have more advanced medical facilities and higher-level skilled staff than level 1 hospitals and, consequently, can provide patients with better standardized pregnancy management. Accordingly, this study found a significant difference in postterm birth rates among the regions and among different levels of hospitals. A lower risk of postterm births was observed in the eastern region and level 3 hospitals. These results indicate that advanced pregnancy management may partially reduce the risk of postterm births. Additionally, women in the eastern region and level 3 hospitals have access to pregnancy dating by ultrasound and close fetal surveillance, which may reduce the risk of postterm births.

The associations between some sociodemographic factors and postterm births have been observed in several epidemiological studies. A population-based cohort study using data from the Swedish Medical Birth Register has found that advanced maternal age is a risk factor for postterm births^18^. Similar findings have been reported in other studies^19,24^. However, our study detected that young women had a higher risk of postterm births, which is supported by another population-based cohort study conducted by Zachary and colleagues^16^. These authors found that a maternal age <20 years was associated with an increased risk of postterm births. Additionally, a Danish study conducted by Olesen *et al*. found that maternal age is not correlated with the prevalence of postterm births^17^. Our study also showed that lower levels of maternal education were strongly correlated with an increased risk of postterm births, which is consistent with Zachary and colleagues’ study^16^. However, other studies have reported that no significant correlation exists between maternal education and postterm births^18,19^. The contradictory results from these studies may be caused by differences in data collection and population selection bias. The association between the selected sociodemographic factors and postterm births could also be modified by other important potentially confounding factors, such as maternal obesity and family history, which could greatly affect the occurrence of postterm births.

Many previous studies have found that nulliparity is a risk factor for prolonged pregnancy^17–19^. However, our study detected that primiparous women had a slightly reduced risk of postterm births. This correlation may be related to the extremely high rate of caesarean sections in Chinese women. Due to a variety of factors—such as rapid urbanization, increased hospitalization during childbirth, financial incentives for healthcare providers to perform caesarean deliveries, and maternal requests for caesarean deliveries because of their anxiety about labor, fear of pain, and desire to choose an auspicious delivery date—the rate of caesarean deliveries in China has rapidly increased during past decades^24,27–29^. According to a recent study conducted by Liang and colleagues, the caesarean section rate remained high at 41.1% in 2016, although it has recently declined in China^30^.

Expectedly, obstetric complications were associated with a reduced risk of postterm births. In some cases, women with obstetric complications are more likely to undergo labor induction before 42 weeks of gestation, which could alter a potential postterm birth to a term birth. In our study, women with direct obstetric complications or other diseases had a reduced risk greater than 30% for postterm births. These results are consistent with previous findings^19^. We also found that maternal history of caesarean sections resulted in a reduced risk of postterm births. These women with a history of caesarean sections constitute a high-risk population, and therefore, there is a high likelihood that they will pay more attention to antenatal care and that their fetuses will be closely monitored during pregnancy.

Interestingly, this study revealed that the risk of postterm births decreased as the frequency of antenatal visits increased. In particular, women with no antenatal visits had double the risk of postterm births. Several reasons can explain this correlation. First, women with more frequent antenatal visits, particularly during the first trimester, are likely to obtain pregnancy dating through ultrasound rather than rely on the last menstrual period. Second, with more frequent antenatal visits, women have a better chance of receiving advanced pregnancy management and close antenatal fetal monitoring and having access to labor induction before 42 weeks of gestation. The Chinese government recommends five or more antenatal visits in rural areas and eight or more antenatal visits in urban areas, which may have contributed to the difference in postterm birth rates among the different regions.

In our analysis, the proportions of women with more than six antenatal visits increased yearly between 2012 and 2016, suggesting an increased probability of obtaining advanced pregnancy management. This could be related to the significant decreasing trend in postterm birth rates. However, most importantly, the postterm birth rates dramatically declined in 2014 and 2016. This could be caused by the relaxation of the one-child policy in November 2013 and the introduction of the two child policy in October 2015^31^. Due to the previously high caesarean section rates, the change in the birth policy could result in more women who previously had caesarean deliveries to give birth a second time. Our analysis shows that the proportions of women with a history of caesarean deliveries increased annually between 2012 and 2016 (data not shown). As reported in our analysis, the postterm birth rates will be decreasing with an increasing number of women with a history of caesarean deliveries. Additionally, the decline in the rates of postterm births was greater in the east than that in the west, suggesting that the birth policy was implemented more effectively in the eastern region.

There are several limitations to our study. First, facility-based sampling may introduce referral bias. The NMNMSS oversampled large hospitals in urban districts, and therefore, we weighed the data using the population distribution in urban districts and rural counties for each region. Second, because we did not collect any information regarding the induction of labor before 42 gestational weeks in the NMNMSS, we cannot assess the effect of labor induction on the prevalence of postterm births in our analysis. Third, some factors, such as pre-pregnancy body mass index and family history, can be associated with postterm births; however, these factors were not included in our analysis.

## Conclusions

In conclusion, postterm births can have a large effect on the long- and short-term health of both the mother and child. Multiple risk factors are associated with the prevalence of postterm births. Identifying these contributing factors could help identify high-risk pregnant women and develop individualized interventions to reduce the risk of postterm births. Interestingly, antenatal visits are a modifiable risk factor, and the increasing frequency of antenatal visits could potentially prevent postterm births during pregnancy. Strengthening antenatal care and management for prolonged pregnancy can be highly valuable, particularly in the western region, for reducing the risk of postterm births.
